# Ethnic and sex differences in skeletal maturation among the Birth to Twenty cohort in South Africa

**DOI:** 10.1136/archdischild-2014-306399

**Published:** 2014-11-19

**Authors:** Tim J Cole, Emily K Rousham, Nicola L Hawley, Noel Cameron, Shane A Norris, John M Pettifor

**Affiliations:** 1 Population Policy and Practice Programme, UCL Institute of Child Health, London, UK; 2 Centre for Global Health and Human Development, School of Sport, Exercise and Health Sciences, Loughborough University, Loughborough, UK; 3 Department of Chronic Disease Epidemiology, School of Public Health, Yale University, New Haven, Connecticut, USA; 4 MRC/Wits Developmental Pathways for Health Research Unit, Faculty of Health Sciences, University of the Witwatersrand, Johannesburg, South Africa

**Keywords:** Adolescent Health, Growth

## Abstract

**Aim:**

To examine ethnic and sex differences in the pattern of skeletal maturity from adolescence to adulthood using a novel longitudinal analysis technique (SuperImposition by Translation And Rotation (SITAR)).

**Setting:**

Johannesburg, South Africa.

**Participants:**

607 boys and girls of black as well as white ethnicity from the Birth to Twenty bone health study, assessed annually from 9 to 20 years of age.

**Outcome measure:**

Bone maturity scores (Tanner–Whitehouse III radius, ulna, and short bones (TW3 RUS)) assessed longitudinally from hand-wrist radiographs were used to produce individual and mean growth curves of bone maturity and analysed by the SITAR method.

**Results:**

The longitudinal analysis showed that black boys matured later by 7.0 SE 1.6 months (p<0.0001) but at the same rate as white boys, whereas black girls matured at the same age but at a faster rate than white girls (by 8.7% SE 2.6%, p=0.0007). The mean curves for bone maturity score consistently showed a midpubertal double kink, contrasting with the quadratic shape of the commonly used reference centile curves for bone maturity (TW3).

**Conclusions:**

Skeletal maturity was reached 1.9 years earlier in girls than boys, and the pattern of maturation differed between the sexes. Within girls, there were no ethnic differences in the pattern or timing of skeletal maturity. Within boys, however, skeletal maturity was delayed by 7 months in black compared with white ethnicity. Skeletal maturation, therefore, varies differentially by sex and ethnicity. The delayed maturity of black boys, but not black girls, supports the hypothesis that boys have greater sensitivity to environmental constraints than girls.

**What is already known on this topic**

- Skeletal maturity is influenced by age, gender and environmental factors, but has been little studied in contemporary populations, particularly in developing communities.
- The timing (tempo) and rate (velocity) of bone maturation during childhood and adolescence determine the age at which adult bone maturity is reached.
- Common modelling techniques used for longitudinal growth data are not applicable to the study of bone maturity from childhood to adulthood.

**What this study adds**

- A novel analysis technique (SuperImposition by Translation And Rotation) is used to create mean curves of bone maturity score by gender and ethnicity.
- Bone maturity growth curves differ by sex, but do not differ by ethnicity (black or white) among South African adolescents.
- Black as well as white girls showed the same pattern and timing of skeletal maturation, whereas black boys were significantly delayed compared with white boys in skeletal maturity.

## Introduction

Skeletal maturity, or ‘bone age’, is a key indicator of biological maturity and reflects progress towards complete fusion of the epiphyses of the long bones. The rate of skeletal maturation is sensitive to environmental influences at both the individual and population levels.^1^ Population differences in the rate and timing of skeletal maturity reflect differences in nutrition, environment, socioeconomic status and genetics.2––5 Secular trends in skeletal maturation have also been observed among populations undergoing rapid social and economic transitions.^6^ ^7^ Skeletal maturity of population samples can be compared with reference samples, most commonly the Tanner–Whitehouse III (TW3) charts,^2^ to compare relative advancement or delay.^8^

Few contemporary studies have been able to chart skeletal maturation longitudinally. The aims of this study are to: describe the skeletal maturation of urban South African adolescents by sex and ethnic group; develop mean growth curves of skeletal maturity and compare these curves with the TW3 reference charts.

## Methods

### Sample

The Birth to Twenty (Bt20) study included singleton children born in Soweto-Johannesburg, South Africa, in 1990.^9^ In 1999, 523 Bt20 participants, (78% African black), were recruited into a bone health substudy. Subsequently, 160 participants (94% white) were recruited in 2000–2001 (total n=683).^9^ ^10^ The analytical sample included 607/683 (88.9%) participants aged 9–20 years with one or more skeletal radiograph(s).

Hand-wrist radiographs were taken annually by radiographers at the Johannesburg Academic Hospital following standard procedures.^2^ Skeletal maturity was assessed by a single observer (NLH) using the TW3 RUS (radius, ulna and short bones) technique.^2^ Individual bone scores were summed to give the RUS bone score, ranging from 0 to 1000 (adult maturity). Intraobserver reliability for the bone age measures was excellent (SE of measurement 0.10 years).

Primary caregivers gave written informed consent for their child to participate. Participants gave their assent annually and consent from 18 years onwards. Ethical approval was obtained from the University of the Witwatersrand Committee for Research on Human Subjects (#M980810) and Loughborough University (R01-P3).

### Statistical analyses

The median age at maturity by sex and ethnicity was estimated cross-sectionally using mixed effects logistic regression, with the dependent variable maturity (RUS bone score=1000) and independent variables age, sex and ethnicity (plus their interaction) as fixed effects, and subject as a random effect. Log transforming age improved the fit. Median age at maturity was estimated as e^−a/b^ with *a,* the intercept and *b,* the log age coefficient; this is the age when half the subjects are immature and half are mature.

Each subject's set of RUS bone scores plotted against age forms a growth or maturation curve. Individual RUS bone scores increase over time until reaching the adult score of 1000. Thus, individual curves differ in terms of the timing and rate of maturation, but not in final size. The SuperImposition by Translation And Rotation (SITAR) method of analysis for longitudinal data^11^ ^12^ is well suited to such growth curves, as it assumes that after adjusting for the timing and rate of maturation, the underlying pattern is the same for all subjects. The method fits a mean curve as a natural regression spline in developmental age, with degrees of freedom chosen to minimise the Bayesian Information Criterion (BIC),^13^ plus two subject-specific random effects defining the timing and rate of maturation.

Conventionally, RUS bone scores are analysed cross-sectionally, for example, TW3 centiles.^2^ With SITAR, the bone scores are analysed as subject-specific curves, where the estimated mean curve reflects the average of the curve shapes seen in individuals, rather than the median score by age. The two forms of curve are usually similar, but they can differ during puberty.^14^ ^15^

Developmental age is assumed to be linearly related to chronological age, and the timing (or *tempo*) and the rate (or *velocity*) of maturation are subject-specific random effects. As there is no variation in size (all individuals reach a bone score of 1000) there is no random effect for size.

The effect of adjusting for tempo is to shift individual curves left/right on the age scale, so as to match the mean curve. Similarly, adjusting for velocity involves shrinking/stretching the age scale to match the mean curve and this has the effect of making the curve steeper or shallower. The net effect of translating and rotating the individual curves is to superimpose them on the mean curve. To measure the goodness-of-fit of the SITAR model, its residual SD is compared with that for the baseline model omitting the random effects.

The data were initially analysed separately by sex and ethnic group (black as well as white), and then joint models were fitted combining first the ethnic groups by sex, and then the sexes. To represent group differences, fixed effects for sex and/or ethnicity were included in the model terms for tempo and velocity, which with their SEs allowed the equality of the group growth patterns to be tested. Log transforming age improved the fit. Thus, the random effects for both tempo and velocity are in fractional units, which multiplied by 100 can be viewed as percentage differences from the mean.^16^ Alternatively, the tempo random effects can be multiplied by mean age to express them in units of years.

For comparison, the SITAR mean curves are plotted against the TW3 centiles for RUS bone score, which were generated from the RUS bone z-scores produced using software of Tanner *et al.*^2^ Models were fitted in the open source statistical software R, using packages lme4, nlme and sitar (available via the Comprehensive R Archive Network website).

## Results

Four thousand five hundred and sixty-five radiographs from 607 subjects aged 9–20 years were scored, with 1–10 scores per subject (median 9, IQR 6–10). The logistic regression model showed that the median age of skeletal maturity was, on average, 1.9 SE 0.2 years earlier in girls than boys (p<0.001); and that girls of black as well as white ethnicity matured at the same age (p=0.7), while black boys matured 6.0 SE 3.2 months later than white boys (p=0.06).

**Figure 1:**
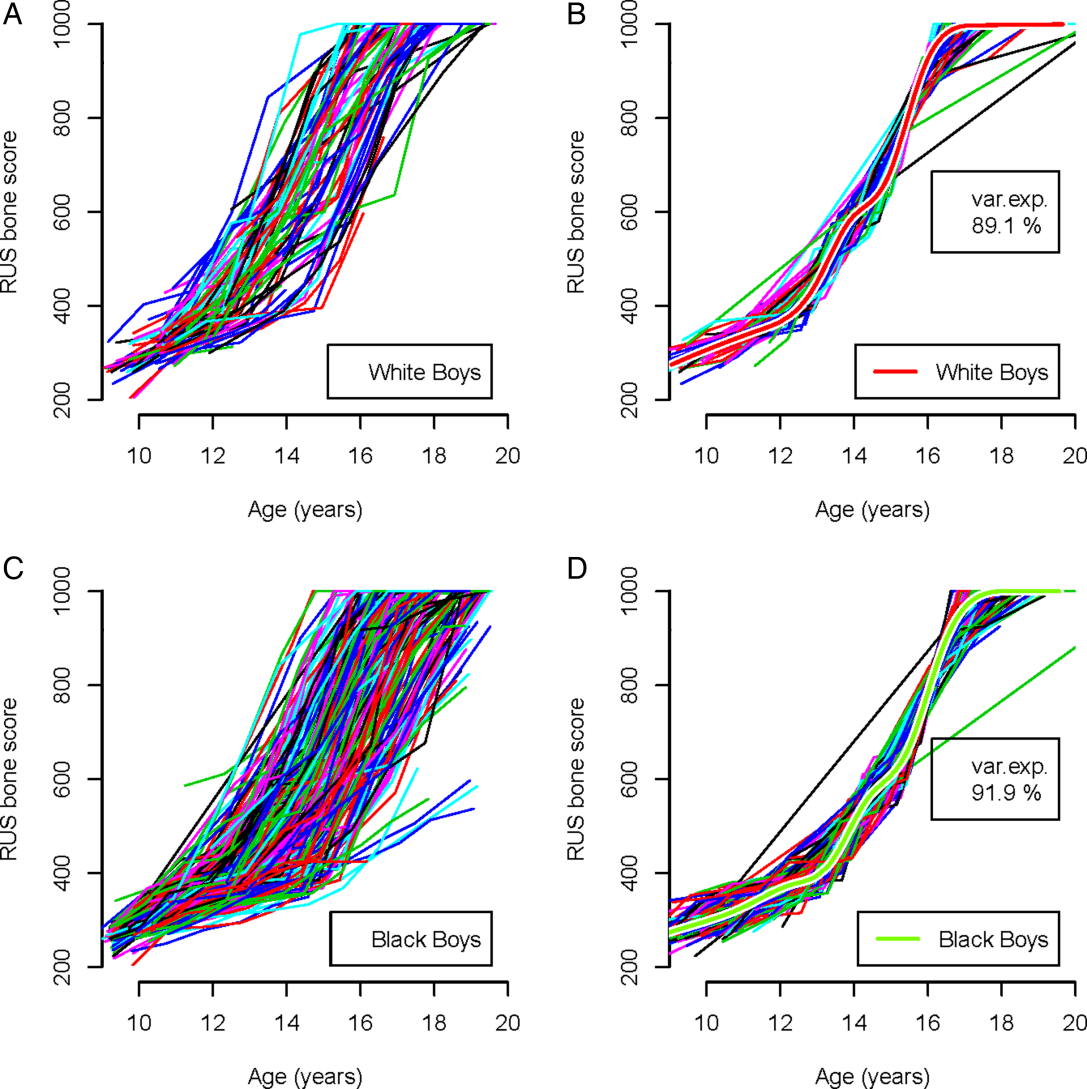
Individual and mean curves of radius, ulna and short bones (RUS) bone maturity score in boys by ethnicity analysed by SuperImposition by Translation And Rotation. (A) White boys: individual unadjusted curves. (B) White boys: individual curves adjusted for tempo and velocity, with the mean adjusted curve. (C) Black boys: individual unadjusted curves. (D) Black boys: individual curves adjusted for tempo and velocity, with the mean adjusted curve.

The SITAR analyses used all the serial bone scores by age. Figure 1 shows the RUS score growth curves for boys by ethnic group, with unadjusted and adjusted curves for individual tempo and velocity. Figure 1A illustrates the range of almost 4 years in maturity timing for white boys and their varying rates of maturation. The range for black boys is wider (figure 1C), due, in part, to their larger numbers. The earlier rising curves tend to be steeper, corresponding to a negative correlation between tempo and velocity. The log transformation for age adjusts for this, as on the log age scale, the curves are more parallel and the tempo–velocity correlation closer to zero.

The SITAR adjustments accounting for differences in tempo and velocity explained 89%–92% of the variance (table 1). The apparently outlying adjusted curves in figure 1B, D are not, in fact, outliers, but curves with a long gap between measurements.

**Table 1:** Median age at maturity, and summary of SITAR analyses of RUS bone score on age fitted to the four groups by sex and ethnicity

	White boys	Black boys	White girls	Black girls
N of subjects/measurements	106/575	214/1803	101/615	186/1572
Median age at maturity (years)	16.5	17.0	15.0	15.0
Spline degrees of freedom	5	7	7	6
Residual SD (bone score units)	29	30	22	23
SD of tempo random effect (%)	6.1	7.9	7.6	7.2
SD of velocity random effect (%)	20	20	22	15
Tempo–velocity correlation	0.2	0.2	–0.2	–0.2
Variance explained (%)	89	92	92	91

RUS, radius, ulna and short bones.

The fitted mean curves are shown superimposed on the adjusted individual curves (figure 1B,D). The mean curves for white and black boys are very similar in shape, becoming steeper in slope after bone score of 400, with a kink at a bone score of 600 and flattening-off approaching a score of 1000.

**Figure 2:**
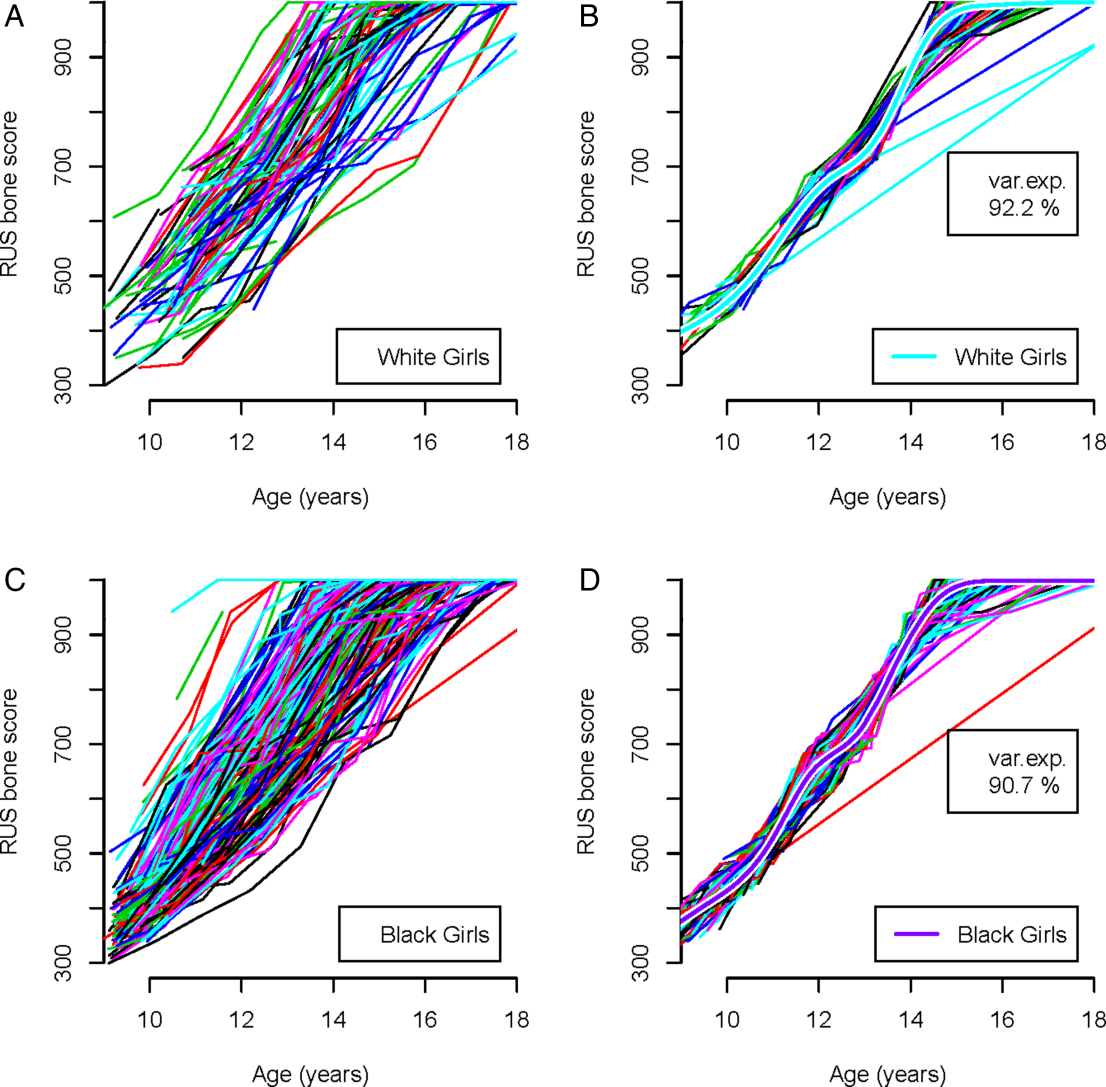
Individual and mean curves of radius, ulna and short bones (RUS) bone maturity score in girls by ethnicity analysed by SuperImposition by Translation And Rotation. (A) White girls: individual unadjusted curves. (B) White girls: individual curves adjusted for tempo and velocity, with the mean adjusted curve. (C) Black girls: individual unadjusted curves. (D) Black girls: individual curves adjusted for tempo and velocity, with the mean adjusted curve.

Figure 2 shows the corresponding curves for girls. The results are similar, with the SITAR model explaining 91%–92% of the variance, and the mean curves in figure 2B, D shaped the same, with kinks at bone scores 400 and 700.

Table 1 summarises the SITAR models for the four groups in figures 1 and 2. The results are very similar by ethnicity for each sex, with residual SDs of 30 bone score units for boys and 23 for girls. The SD for tempo, which indicates the variability in the timing of puberty, is 6%–8%, or multiplied by mean age 14.2 years, SD 0.9–1.1 years. The SD for velocity is larger at 15%–21%. The correlations between tempo and velocity are small, indicating that the log age transformation on which they are based adjusts for the strong negative correlation visible in figures 1 and 2.

Given the similarity of the models by ethnicity within each sex, combined models were fitted to the boys and girls separately, allowing the mean values for tempo and velocity to differ by ethnicity, and they fitted well (BIC for boys 24 844 combined vs 24 871 separate; for girls 21 742 combined vs 21 780 separate). However, a model combining the sexes fitted poorly (BIC 46 919 combined vs 46 586 separate), showing that the two mean curves were different in shape and should not be combined.

**Figure 3:**
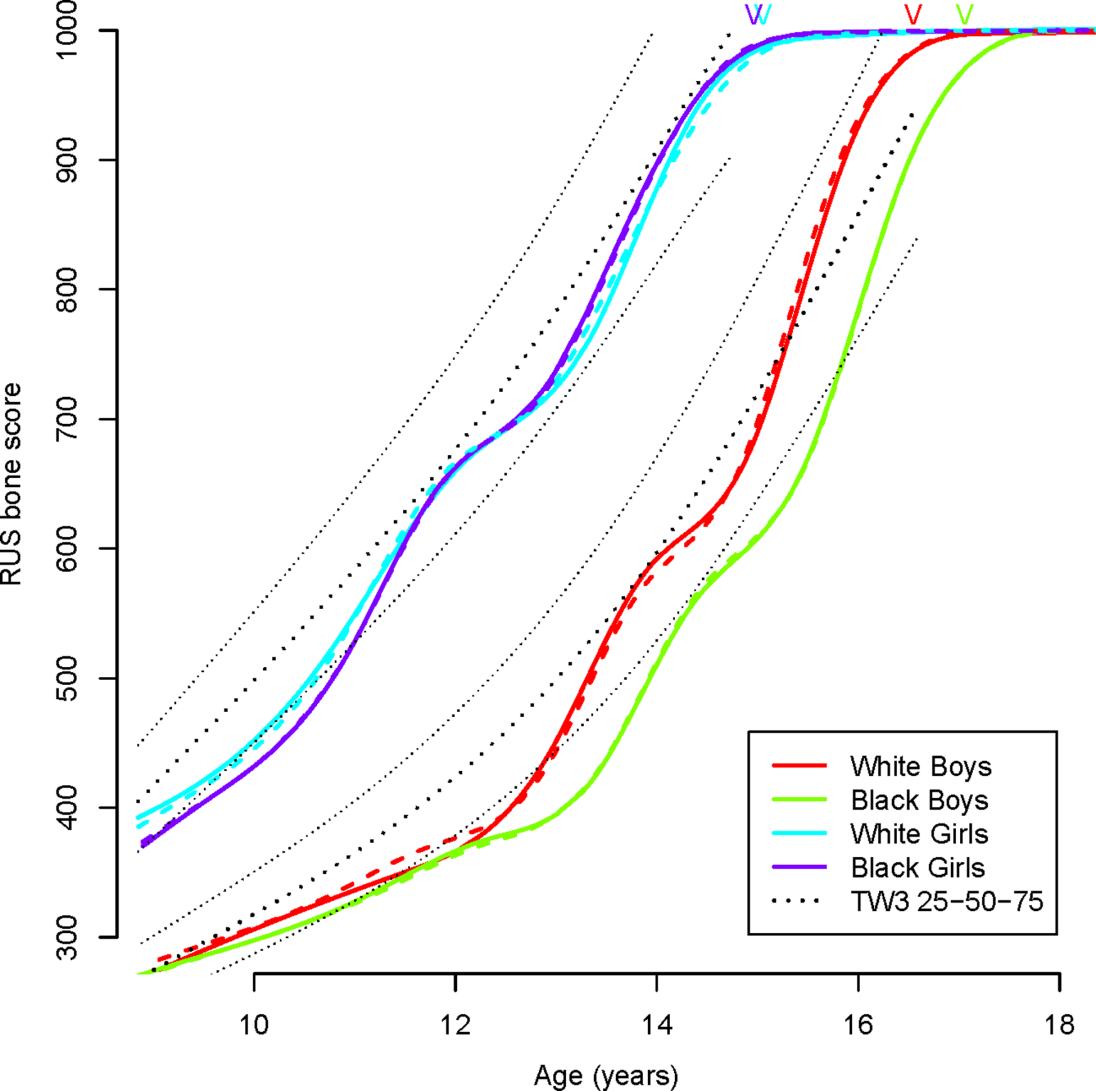
Mean curves by sex and ethnicity of radius, ulna and short bones (RUS) bone maturity score in the Birth to Twenty subsample compared with the Tanner–Whitehouse III (TW3) reference (25th, 50th and 75th centiles).^2^ V indicates the median age of attaining adult bone maturity (RUS bone maturity score of 1000) in each group.

Figure 3 compares the mean curves for the four sex-ethnic groups, as fitted separately (dotted lines) and jointly within sex (solid lines). Also included are the group median ages at maturity from table 1 and the TW3-RUS 25th, 50th and 75th centiles (dotted lines). The pairs of curves by sex are closely similar, confirming that the underlying curve shapes are the same for white as well as black children. The boys’ curves are parallel but separated in age, corresponding to an ethnic difference in timing (tempo). The girls’ curves lie close together, but are steeper for black girls. Table 2 summarises these differences as SITAR-fixed effects for tempo and velocity in the black compared with white children: tempo in black boys was 7 months later than in white boys, while velocity in black girls was 9% greater than in white girls; both highly significant. By contrast, the differences in boys’ velocity or in girls’ tempo were tiny. The tempo difference in boys was similar to that derived from the logistic regression analysis (6.0 SE 3.2 months) but with an SE half the size, emphasising the advantage of SITAR using all the data.

**Table 2:** Summary of SITAR fixed effects for black ethnicity compared with white ethnicity, by sex

	Regression coefficient	SE	p Value
Boys			
Tempo (months)	7.0	1.6	<0.0001
Velocity (%)	−1.0	3.0	0.7
Girls			
Tempo (months)	0.0	1.8	>0.9
Velocity (%)	8.7	2.6	0.0007

Figure 3 shows that girls of both ethnicities lie between the 25th and 50th centiles from 11 years onwards. White boys lie between the 25th and 75th centiles from 13 years onwards, while black boys reach the 25th centile only after 14 years and remain below the 50th centile, demonstrating that their maturity is delayed relative to the TW3-RUS references.

Figure 3 also shows a consistent difference in shape between the SITAR growth curves with a double-kink and the quadratic curves of the TW3-RUS reference centiles.

## Discussion

This study presents unique longitudinal data on skeletal maturation in adolescents from a middle-income country. Moreover, the study provides mean growth curves for RUS bone scores which permit the analysis of sex and ethnic differences in patterns of skeletal maturation. The results show that black boys mature later than white boys whereas black as well as white girls mature at the same age. A longitudinal analysis clarifies how this pattern emerges; skeletal maturation in black girls starts to accelerate later than in white girls, but develops faster, whereas in black boys, skeletal maturation starts to accelerate later and then develops at a similar rate.

The differing patterns of skeletal maturity in the two ethnic groups by sex are intriguing. In all populations, girls are more skeletally mature than boys from birth onwards and reach adult bone maturity, on average, 2 years earlier than boys (1.9 years here).^2^ Similarly, lower socioeconomic status is generally associated with delayed bone maturity scores compared with higher socioeconomic status.^1^ Adverse environmental circumstances affect the growth of males relatively more than females in all species,^17^ ^18^ and skeletal maturity is thought to be delayed in boys more than in girls in unfavourable environments.^2^ This study provides new evidence that black as well as white South African girls are broadly synchronised in their timing of skeletal maturity, whereas black boys are appreciably delayed compared with white boys. Previous analyses of pubertal development in the bone health subsample found no differences in the age at initiation of puberty by ethnic group or sex^19^ and no ethnic differences in median age at menarche (12.4 and 12.5 years, black girls and white girls respectively.^20^ Black ethnicity is associated with advanced puberty in some contexts^21^ ^22^ but not here.^19^ ^20^ In general, the processes of secondary sexual development and skeletal development are only weakly associated, whereas pubertal events (eg, menarche) and skeletal development are strongly associated.^23^ ^24^

This is the first study to produce mean growth curves for skeletal maturity by sex and ethnicity. The mean curves are closely similar in the two ethnic groups by sex, indicating that the curve shape is robust. The curves are notably different from longitudinal growth curves of height and weight in puberty^12^ ^25^ ^26^ where the velocity curves have a single peak (peak velocity). The growth curves in figures 1–3 increase monotonically with age but display a double kink (at RUS score of 600 in boys and 700 in girls). This generates a velocity curve with two peaks rather than one, thus it is not possible to identify a unique age at peak velocity.

What does this pattern mean? The curve shapes are likely to be genuine for two reasons; first, the same pattern emerged independently in all four groups, implying that this may be universal; and second, the complexity of each curve's shape was data-driven—the number of degrees of freedom for the regression spline was chosen to minimise the BIC, which penalises the deviance (goodness-of-fit) for each extra degree of freedom used. Thus, the models all fitted appreciably better with the extra degrees of freedom, implying that the *individual subject* curves also have the same underlying shape.

All four curves are based on the TW3 scoring system, which converts the radiographic appearance of the radiographs to RUS bone scores. Unlike height or weight, the scores are on a constructed scale, which will not necessarily represent growth as a smoothly changing process. Figure 3 shows that the longitudinal growth curves are different in shape from the cross-sectional TW3 centiles, suggesting that the SITAR analysis has teased out more detail in the pattern of bone maturation and that the TW3 centiles are to an extent oversmoothed.

The findings also cast light on individual variation in skeletal maturation. The SITAR models fitted consistently better when using log age rather than age. This indicates a multiplicative age scale, where individuals whose maturation is advanced have a foreshortened (or shrunken) scale, while delayed maturers have an extended (stretched) scale. This corresponds to individuals starting to mature at birth and continuing to mature relatively quickly or slowly through childhood until reaching maturity. This is analogous to Peto's ‘horse-racing effect’,^27^ where the horse leading the race is running the fastest. Consequently, advanced maturers have an early puberty and also a faster passage through puberty. In the present study, this pattern is most apparent in the girls.

A particular strength of this study is the availability of longitudinal bone maturity scores in two ethnic groups of children and adolescents with all ratings undertaken by a single observer.

In conclusion, the study has shown that the pattern of skeletal maturation in South African adolescents is similar in white and black girls, but delayed by 7 months in black boys compared with white boys. The fact that skeletal maturity was delayed in black boys, but not in black girls, supports the hypothesis that boys are more susceptible to delays in growth and maturation in unfavourable environments.^18^ ^28^
